# Safely meeting global salmon demand

**DOI:** 10.1038/s41538-018-0025-5

**Published:** 2018-09-27

**Authors:** Gary B. Smejkal, Srikanth Kakumanu

**Affiliations:** Focus Proteomics, 46 Derry Road, Hudson, NH 03051 USA

**Keywords:** Animal physiology, Environmental impact

For the first time in human history, global farmed fish production exceeds beef production. This from a civilization that has propagated cattle to nearly twice its own biomass and which utilizes over a third of the planet’s ice-free land surface for agriculture.^1,2^ In 2016, salmon surpassed tuna to become the second most-consumed seafood in the United States, second only to shrimp.^3^

It is estimated that 91% of the seafood consumed in the United States is imported, of which nearly half is produced through aquaculture.^4^ The United States Food and Drug Administration (FDA) inspects only approximately 2% of imported seafood, of which less than 0.1% is tested for banned chemical residues.^5^ In 2017, the FDA inspected 86 samples from 379,000 tons of imported salmon.^6^

Norway is the global leader in salmon aquaculture, producing more salmon than the United Kingdom, Chile, Canada, and the Faroe Islands combined.^7^ The salmon biomass in Norway twice outweighs the entire human population in that country.^8^ The source of the salmon is important, as farmed salmon have been shown to contain higher levels of persistent organic pollutants than wild caught salmon. For example, farmed Atlantic salmon from Scotland and the Faroe Islands were nearly ten times higher in pollutants such as polychlorinated biphenyls, dioxins, and the organochlorine pesticides dieldrin and toxaphene than in wild-caught Pacific salmon from Alaska.^9^ This increase was linked to the bioaccumulation of persistent organic pollutants from the pelagic fish used in salmon feed.^10^ Over the past decade, the use of non-pelagic feeds from terrestrial sources in Norwegian salmon farms has produced salmon with lower concentrations of persistent organic pollutants and mercury than wild-caught Atlantic salmon from the same region, but with lowered levels of the beneficial docosahexanoic acid in the farmed salmon.^11^

Under the U.S. Food Safety Modernization Act, increased inspections by the FDA and the U.S. Department of Agriculture Food Safety Inspection Service (FSIS) have resulted in record numbers of seafood rejections, clearly demonstrating the persistence of banned chemicals in aquaculture, both foreign and domestic. In 2016, nearly 15 tons of frozen swai imported from Vietnam and distributed in the United States were recalled for failure to meet FSIS requirements.^12^ In 2018, more than 34 tons of farm-raised Siluriformes from Mississippi that contained leucomalachite green were recalled.^13^

Researchers have previously reported that over 25% of fish imported from Asia that were sampled from Colorado and North Carolina supermarkets contained significantly higher levels of formaldehyde than fish from domestic sources.^14,15^ While formaldehyde naturally occurs in trace amounts in most living tissues, its formation is accelerated in decomposing fish tissues by the reduction of trimethylamine oxide.^16^ In 2015, Alaskan fisheries reported record catches of wild salmon,^17^ of which an estimated 70% was exported.^18^ Some of this fish is processed in China, then imported back to the United States where it can be labeled “wild caught Alaskan salmon”.^19^ The FDA does not routinely inspect seafood imports for formaldehyde.

In aquaculture, open net cage systems concentrate large populations of fish in confined areas making them particularly susceptible to parasites and the rapid spread of disease. Sea lice infestations (*Lepeophtheirus salmonis* and *Caligus elongates*) represent one of the most significant disease problems currently affecting salmon aquaculture. The avermectin pesticide emamectin benzoate (EMB) is approved for use in treating sea lice in farmed salmon and trout in Canada, Norway, Chile and the United Kingdom, but has not received FDA approval in the United States.^20^ The prescribed dosage of EMB for salmon is 50 ug/kg body mass per day for seven days, which is effective against all parasitic stages of sea lice. Fish can be treated up to three times per year with a maximum of five treatments in two years.^21^ The maximum allowable residue limit of emamectin B1a residue is 100 ug/kg in salmon muscle intended for human consumption and 1000 ug/kg in skin.^22–24^ Emamectin B1a accumulates in the liver and residual levels as high as 9000 ug/kg have been observed one week after treatment has ceased.^25^ The half-life of EMB is 11.3 days and a withdrawal period (the period from the last treatment to when the fish can be harvested for human consumption) of 60 days has been recommended.^26^ However, a seven day withdrawal period is observed in Norway,^27^ while no withdrawal period is required in Scotland and Chile.^28^

As an alternative to pesticides, the FDA has approved garlic for the control of parasites in aquaculture. Garlic derivatives have been shown to significantly lower sea lice in salmonids^29^ and monogenean parasites in other fish.^30–32^

The terms sustainable and responsible are not synonymous. Larger salmon farms can sustain over 720,000 fish, roughly the biomass equivalent of 360 Indian elephants, usually in open net cages from which waste is released directly into the ocean. In 2000, Scottish salmon farms collectively discharged 7500 metric tons of nitrogen, comparable to the annual sewage produced by 3.2 million people, and more phosphorous than produced by the entire Scottish population of 5.3 million people.^33^

Concerns over the safety of farmed salmon have increased the demand for wild-caught Alaskan salmon, driving higher prices and even counterfeiting. Genetic analysis of 466 salmon samples collected from 2010 through 2014 showed that 14% were mislabeled.^34^ The most common form of mislabeling was farmed Atlantic salmon being sold as wild salmon. During the winter months, when wild salmon is out of season, mislabeling of 43% was reported.
